# The effects of normobaric and hyperbaric oxygenation on MRI signal intensities in *T_1_*-weighted, *T_2_*-weighted and FLAIR images in human brain

**DOI:** 10.2478/raon-2023-0043

**Published:** 2023-09-04

**Authors:** Vida Velej, Ksenija Cankar, Jernej Vidmar

**Affiliations:** Institute of Physiology, Faculty of Medicine, University of Ljubljana, Ljubljana, Slovenia; Kranj Community Health Center, Gorenjska Basic Healthcare, Kranj, Slovenia; Institute of Radiology, University Medical Center Ljubljana, Ljubljana, Slovenia

**Keywords:** hyperbaric oxygen, normobaric oxygen, magnetic resonance, human brain

## Abstract

**Background:**

Dissolved oxygen has known paramagnetic effects in magnetic resonance imaging (MRI). The aim of this study was to compare the effects of normobaric oxygenation (NBO) and hyperbaric oxygenation (HBO) on human brain MRI signal intensities.

**Patients and methods:**

Baseline brain MRI was performed in 17 healthy subjects (mean age 27.8 ± 3.2). MRI was repeated after exposure to the NBO and HBO at different time points (0 min, 25 min, 50 min). Signal intensities in *T*_1_-weighted, *T*_2_-weighted images and fluid attenuated inversion recovery (FLAIR) signal intensities of several intracranial structures were compared between NBO and HBO.

**Results:**

Increased *T*_1_-weighted signal intensities were observed in white and deep grey brain matter, cerebrospinal fluid (CSF), venous blood and vitreous body after exposure to NBO as well as to HBO compared to baseline (Dunnett's test, *p* < 0.05) without significant differences between both protocols. There was also no significant difference in *T*_2_-weighted signal intensities between NBO and HBO. FLAIR signal intensities were increased only in the vitreous body after NBO and HBO and FLAIR signal of caudate nucleus was decreased after NBO (Dunnett's test, *p* < 0.05). The statistically significant differences in FLAIR signal intensities were found between NBO and HBO (paired t-test, *p* < 0.05) in most observed brain structures (paired t-test, *p* < 0.05).

**Conclusions:**

Our results show that NBO and HBO alters signal intensities *T*_1_-weighted and FLAIR images of human brain. The differences between NBO and HBO are most pronounced in FLAIR imaging.

## Introduction

Magnetic resonance imaging (MRI) of brain is a superior soft-tissue contrast method that is used for the assessment of a numerous neurological conditions such as multiple sclerosis and headaches, and used to characterize strokes and space-occupying lesions. Basic MRI brain screen protocol is a simple non-contrast MRI comprising a group of basic MRI sequences when imaging the brain in cases of no particular condition is being sought (e.g. headache). The protocol is designed to obtain a good general overview of the brain. A standard screening protocol might include *T*_1_ weighted imaging for anatomical overview, *T*_2_ weighted imaging to evaluate basal cisterns, ventricular system and subdural spaces, and good visualization of flow voids in vessels, fluid attenuated inversion recovery imaging (FLAIR) to assess white-matter, diffusion weighted imaging (DWI) for multiple possible purposes (from the identification of ischemic stroke to the assessment of active demyelination).

Dissolved oxygen can be used as a contrast agent in MRI due to the paramagnetic properties of the dioxygen molecule O_2_^1^. Since O_2_ as well as hydroxyl and superoxide radicals contain unpaired electrons, they exhibit paramagnetic effect and may shorten the spin-lattice relaxation time (*T*_1_) in magnetic resonance imaging (MRI).^2,3,4,5,6^ *T*_1_ relaxation times shortening under the influence of increased partial pressure of oxygen (pO_2_) in the inspired gas mixture is called tissue-oxygen-level-dependent effect (TOLD). It was observed in many tissues: arterial blood, myocardium, spleen, skeletal muscles, renal cortex, liver and fat.^4,7,8,9^ TOLD effect was also detected in brain parenchyma (grey and white matter) and cerebrospinal fluid (CSF).^10,11,12,13,14,15,16^ In addition, pO_2_ increase also affects spin-spin relaxation time (*T*_2_)^13,17^; however, results of available studies on the effect of pO_2_ on *T*_2_ relaxation times are controversial.^7,12^ FLAIR images of healthy volunteers also showed increased CSF signal intensity during 100% oxygen breathing.^18^

Concentration of dissolved oxygen is directly proportional to its partial pressure, pO_2_.^19^ High pO_2_ values in arterial blood as well as in brain parenchyma can be achieved with normobaric 100% oxygenation (NBO) compared to breathing normobaric air (NBA). Hyperbaric 100% oxygenation (HBO) causes a more pronounced increase of arterial and brain pO_2_ compared to NBO as well as augments production of reactive oxygen species (ROS).^20,21,22^ In few animal studies, it has been already observed that HBO had a more pronounced effect on *T*_1_ and *T*_2_ relaxation times compared to breathing NBO or NBA.^11,23^

To our knowledge, no human studies were performed studying the effect of HBO on MRI signal intensities. The aim of this study was to compare the effects of HBO and NBO on MRI signal intensities (e.g. *T*_1_, *T*_2_ and FLAIR).

## Patients and methods

The study was approved by The National Ethics Committee (No. 0120-203/2019/4). Research was conducted at the Institute of Physiology (University of Ljubljana, Faculty of Medicine). Informed consent was obtained from each subject. 17 healthy volunteers (12 males and 5 females), age 20–40 years (mean age 27.8 ± 3.2), were enrolled in the study. Exclusion criteria were: history of a neurological disorder, non-MRI-compatible devices, a lung disease with FEV1/FVC < 60% and/or emphysema and/or pneumothorax, history of middle ear trauma or disease, therapy with platinum complexes, doxorubicin, bleomycin, disulfiram of mafenide acetate, pregnancy or claustrophobia.

### Study protocol

**FIGURE 1.**
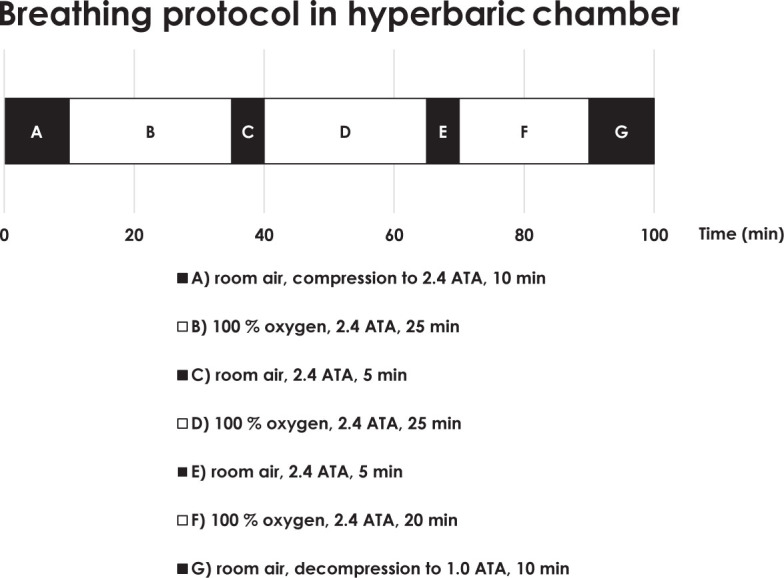
Hyperbaric oxygenation (HBO) protocol.

MRI examination was performed before oxygen breathing protocol (baseline state), after HBO and after NBO with subsequent MRI on separate visits. NBO protocol was performed using a non-rebreather oxygen mask connected to a large reservoir supplied by 100% oxygen for 70 minutes. HBO protocol was performed in multiplace hyperbaric chamber (Kovinarska P&P, Slovenia) at 2.4 ATA with breathing of 100% oxygen for 70 minutes as shown in Figure 1. After each oxygen breathing protocol (NBO or HBO), MRI examination was repeated three times, i.e. immediately after the end of HBO or NBO, after 25 min and after 50 min.

### MR image acquisition

The MRI imaging was performed on a 3T MRI system (TX Achieva Philips Netherlands) with the use of a 32-channel head coil. The MR examination consisted of:

- *T*_1_ spin echo (SE) imaging in the transversal plane with imaging parameters: repetition time (TR) 1026 ms, echo time (TE) 10 ms, filed of view (FOV) 230 × 183 mm, matrix 256 × 163, voxel 0.9 × 1.12 mm, slice thickness 4 mm, gap 1 mm, number of slices 29, number of signals averaged (NSA) 2 with approximate duration of 5 min 38 s
- *T*_2_ turbo spin echo (TSE) imaging in the transversal plane with imaging parameters: TR 9179 ms, TE 100 ms, FOV 230 × 185 mm, matrix 384 × 229, voxel 0.6 × 0.75 mm, slice thickness 3 mm, gap 0 mm, number of slices 50, NSA 3, sensitivity (SENS) 1.7 with approximate duration of 4 min 55 s.
- FLAIR in transversal plane: TR 11000 ms, TE 125 ms, TI 2800 ms, FOV 230 × 183 mm, matrix 328 × 185, voxel 0.7 × 0.93 mm, slice thickness 3 mm, gap 1 mm, number of slices 36, NSA 2, SPIR technique, SENS 2 with approximate duration: 3 min 51 s.

The total MRI scanning time during one MR examination was 25 min.

### MR data and statistical analysis

The MRI images were analysed using ImageJ free image analysis software (National Institutes of Health, USA). Mean signal values in distinct regions of interest (ROI) on *T*_1_-weighted, *T*_2_-weighted and FLAIR images were obtained: frontal white matter, thalamus, caudate nucleus, putamen, hippocampus, superior sagittal sinus, vitreous body and cerebrospinal fluid (CSF).

Statistical analysis was performed using SigmaPlot 14.0 (Systat Software, Inc., USA). The signal intensities after NBO or HBO were compared to the baseline values. Shapiro-Wilk test and Brown-Forsythe were used to check for normality and equal variance. One-way repeated measurements analysis of variance (RM ANOVA) was used to test for differences between signal intensities before, immediately, 25 min and 50 min after NBO or HBO. In cases when Shapiro-Wilk or Brown-Forsythe test failed, Friedman RM ANOVA on Ranks was performed. If RM ANOVA showed statistically significant differences between groups of data, Dunnett's method for multiple comparisons was used to compare signal intensities at three time points after oxygen breathing protocol with baseline values. Additionally, RM ANOVA or Friedman RM ANOVA on Ranks was used to check for differences in signal values of each ROI in *T*_1_-weighted, *T*_2_-weighted and FLAIR images between baseline signal values and values after HBO/NBO at each time point (0 min, 25 min, 50 min). The signal intensity changes in *T*_1_-weighted, *T*_2_-weighted and FLAIR images compared to baseline in each ROI at each time point (0 min, 25 min, 50 min) after NBO and HBO were calculated. Paired t-test was used to compare the signal intensity changes at each time point between NBO and HBO. In cases when Shapiro-Wilk normality test failed, Wilcoxon signed rank test was performed. The α level was set at *p* < 0.05 for all statistical significances.

## Results

The results of *T*_1_-weighted signal intensities before, immediately, 25 min and 50 min after NBO or HBO are presented in Table 1. After NBO there was a statistically significant increase in *T*_1_-weighted signal intensity in all studied structures except for vitreous body and putamen (RM ANOVA, Dunnett's test, *p* < 0.05). In contrast, after HBO we observed a significant increase in *T*_1_-weighted signal intensities except for the superior sagittal sinus and CSF (Dunnett's test, *p* < 0,05). *T*_1_-weighted signal intensity was significantly higher immediately (0 min) as well as 25 min after the end of the HBO compared to *T*_1_-weighted signal intensity immediately and 25 min after NBO in vitreous body (paired t-test, *p* < 0.05). In contrast, there was no difference in signal intensities in *T*_1_-weighted images between HBO and NBO after 50 min.

**TABLE 1.** Comparison of *T*_1_-weighted signal intensities before, immediately, 25 min and 50 min after normobaric oxygenation (NBO) **(A)** and hyperbaric oxygenation (HBO) **(B)** (mean ± standard deviation)

**A) NBO**					

**Structure**	Baseline	0 min	25 min	50 min	p
**Frontal white matter**	770.1 ± 251.2	791.0 ± 238.4	837.3 ± 328.4	851.3 ± 337.7^*^	**0.044**
**Thalamus**	827.9 ± 275.6	846.7 ± 244.7	894.6 ± 338.4	914.7 ± 356.5^*^	**0.038**
**Head of caudate nucleus**	731.9 ± 238.3	753.8 ± 227.0	798.7 ± 318.8	820.9 ± 331.3^*^	**0.023**
**Putamen**	800.2 ± 257.0	814.2 ± 239.2	860.7 ± 333.8	878.1 ± 350.2	0.059
**Hippocampus**	701.6 ± 232.0	712.1 ± 211.3	751.8 ± 285.5	771.6 ± 303.8^*^	**0.038**
**Superior sagittal sinus**	818.0 ± 375.6	748.2 ± 252.1	778.3 ± 288.6	860.9 ± 304.8	**0.019**
**Cerebrospinal fluid**	356.6 ± 126.8	362.7 ± 108.5	396.6 ± 152.9^*^	397.7 ± 163.0^*^	**0.010**
**Vitreous body**	281.3 ± 89.6	288.0 ± 92.1	296.8 ± 115.0	302.5 ± 116.3	0.264

**B) HBO**					

**Structure**	Baseline	0 min	25 min	50 min	p
**Frontal white matter**	770.1 ± 251.2	834.7 ± 133.0	860.9 ± 158.9	886.6 ± 184.7^*^	**0.026**
**Thalamus**	827.9 ± 275.6	900.8 ± 147.1	933.3 ± 169.1	957.1 ± 193.9^*^	**0.004**
**Head of caudate nucleus**	731.9 ± 238.3	794.9 ± 124.4	819.9 ± 142.4	846.2 ± 173.7	**0.013**
**Putamen**	800.2 ± 257.0	867.1 ± 134.9	893.9 ± 154.6	922.5 ± 185.1^*^	**0.007**
**Hippocampus**	701.6 ± 232.0	764.8 ± 124.1	787.2 ± 142.1	807.5 ± 163.2	**0.044**
**Superior sagittal sinus**	818.0 ± 375.6	848.0 ± 227.8	911.9 ± 310.7	907.6 ± 335.0	0.127
**Cerebrospinal fluid**	356.6 ± 126.8	396.4 ± 60.2	400.7 ± 73.7	413.0 ± 98.9	0.256
**Vitreous body**	281.3 ± 89.6	361.2 ± 63.0^*^	335.9 ± 69.6	349.4 ± 82.8	**0.040**

* statistically significant difference compared to baseline value at p < 0.05; NBO = 100 % normobaric oxygen, HBO = 100 % hyperbaric oxygen

The results of *T*_2_-weighted signal intensities before, immediately, 25 min and 50 min after NBO or HBO are presented in Table 2. *T*_2_-weighted signal intensities were increased only in frontal white matter and thalamus after NBO and in the superior sagittal sinus and vitreous body after HBO (Dunnett's test, *p* < 0.05). There was also no significant difference in *T*_2_-weighted signal intensities between HBO and NBO.

**TABLE 2.** Comparison of *T*_2_-weighted signal intensities before, immediately, 25 min and 50 min after normobaric oxygenation (NBO) **(A)** and hyperbaric oxygenation (HBO) **(B)** (mean ± standard deviation)

**A) NBO**					

**Structure**	Baseline	0 min	25 min	50 min	p
**Frontal white matter**	362.5 ± 33.1	367.8 ± 32.8	397.1 ± 80.4^*^	389.0 ± 51.3^*^	**0.007**
**Thalamus**	480.6 ± 49.6	485.1 ± 63.5	521.0 ± 114.8	508.0 ± 61.7	**0.022**
**Head of caudate nucleus**	621.8 ± 64.8	637.9 ± 54.0	673.6 ± 139.8	656.7 ± 91.4	0.631
**Putamen**	514.0 ± 57.0	527.7 ± 50.7	552.3 ± 103.4	542.2 ± 64.1	0.073
**Hippocampus**	694.9 ± 71.9	712.6 ± 83.5	756.9 ± 190.3	734.0 ± 98.2	0.281
**Superior sagittal sinus**	41.3 ± 6.8	42.7 ± 8.6	45.7 ± 12.5	45.0 ± 11.3	0.317
**Cerebrospinal fluid**	1996.8 ± 143.6	2059.7 ± 203.3	2171.2 ± 522.0	2107.9 ± 274.3	0.318
**Vitreous body**	1404.8 ± 114.8	1499.4 ± 159.1	1585.5 ± 396.5	1520.9 ± 224.1	0.080

**B) HBO**					

**Structure**	Baseline	0 min	25 min	50 min	p
**Frontal white matter**	362.5 ± 33.1	359.4 ± 26.7	367.0 ± 35.5	375.1 ± 44.7	0.223
**Thalamus**	480.6 ± 49.6	473.6 ± 34.4	479.3 ± 33.3	489.1 ± 59.1	0.365
**Head of caudate nucleus**	621.8 ± 64.8	617.8 ± 47.3	620.9 ± 50.0	639.5 ± 77.1	0.390
**Putamen**	514.0 ± 57.0	510.9 ± 37.1	514.6 ± 39.8	532.9 ± 65.4	0.256
**Hippocampus**	694.9 ± 71.9	695.9 ± 57.2	688.1 ± 38.4	713.4 ± 81.4	0.378
**Superior sagittal sinus**	41.3 ± 6.8	48.3 ± 14.0^*^	47.4 ± 11.8	48.0 ± 15.0	**0.047**
**Cerebrospinal fluid**	1996.8 ± 143.6	1972.3 ± 79.2	1984.8 ± 102.3	2027.9 ± 195.4	0.482
**Vitreous body**	1404.8 ± 114.8	1524.0 ± 114.7^*^	1529.5 ± 143.2^*^	1530.9 ± 189.8^*^	0.001

* statistically significant difference compared to baseline value at p < 0.05; NBO = 100 % normobaric oxygen; HBO = 100 % hyperbaric oxygen

The results of FLAIR signal intensities before, immediately, 25 min and 50 min after NBO or HBO are presented in Table 3. FLAIR signal intensities were increased only in the vitreous body after NBO and HBO, signal of caudate nucleus was decreased after NBO (Dunnett's test, *p* < 0.05).

**TABLE 3.** Comparison of FLAIR signal intensities before, immediately, 25 min and 50 min after normobaric oxygenation (NBO) **(A)** and hyperbaric oxygenation (HBO) **(B)** (mean ± standard deviation)

**A) NBO**					

**Structure**	Baseline	0 min	25 min	50 min	p
**Frontal white matter**	731.7 ± 77.0	700.8 ± 89.8	713.7 ± 125.1	717.4 ± 128.7	0.615
**Thalamus**	897.1 ± 101.7	851.5 ± 96.7	868.6 ± 164.3	861.5 ± 153.6	0.399
**Head of caudate nucleus**	1119.2 ± 131.9	1083.0 ± 129.4	1070.0 ± 213.0	1030.0 ± 172.5^*^	**0.039**
**Putamen**	928.1 ± 116.5	875.8 ± 129.0	893.2 ± 184.3	893.0 ± 171.4	0.354
**Hippocampus**	1216.2 ± 135.7	1162.1 ± 144.8	1173.5 ± 223.0	1174.1 ± 239.1	0.490
**Superior sagittal sinus**	91.2 ± 23.6	84.6 ± 23.5	78.2 ± 27.2	86.0 ± 33.2	0.299
**Cerebrospinal fluid**	139.6 ± 33.3	140.5 ± 39.8	147.0 ± 51.8	157.8 ± 53.2	0.228
**Vitreous body**	127.2 ± 29.4	170.4 ± 49.1^*^	157.3 ± 45.0^*^	147.3 ± 45,9	**0.002**

**B) HBO**					

**Structure**	Baseline	0 min	25 min	50 min	p
**Frontal white matter**	731.7 ± 77.0	755.8 ± 113.1	794.7 ± 135.2	782.2 ± 115.3	0.508
**Thalamus**	897.1 ± 101.7	927.1 ± 104.6	691.3 ± 127.3	949.7 ± 110.5	0.973
**Head of caudate nucleus**	1119.2 ± 131.9	1180.6 ± 183.3	1208.0 ± 175.8	1190.8 ± 152.3	0.508
**Putamen**	928.1 ± 116.5	958.3 ± 143.4	993.0 ± 148.2	978.9 ± 133.4	0.567
**Hippocampus**	1216.2 ± 135.7	1269.4 ± 171.3	1294.4 ± 184.7	1286.7 ± 160.4	0.771
**Superior sagittal sinus**	91.2 ± 23.6	93.4 ± 25.8	105.1 ± 30.5	106.4 ± 37.0	0.193
**Cerebrospinal fluid**	139.6 ± 33.3	134.5 ± 27.0	139.4 ± 20.2	138.3 ± 21.8	0.909
**Vitreous body**	127.2 ± 29.4	691.4 ± 142.9^*^	523.6 ± 122.9^*^	378.1 ± 88.8	**< 0.001**

* statistically significant difference compared to baseline value at p < 0.05; NBO = 100 % normobaric oxygen; HBO = 100 % hyperbaric oxygen

The statistically significant differences in FLAIR signal intensities were found between NBO and HBO (paired t-test, *p* < 0.05) in caudate nucleus, thalamus, hippocampus and vitreous body at each time point (0 min, 25 min, 50 min). In addition, the differences were also observed between NBO in HBO in putamen and frontal white matter at 0 min and 25 min and in superior sagittal sinus at 25 min (paired t-test, *p* < 0.05).

## Discussion

In the present study we observed increased signal intensity in *T*_1_-weighted imaging in frontal white matter, thalamus, caudate nucleus and hippocampus after NBO as well as HBO, in superior sagittal sinus and CSF after NBO and in vitreous body and putamen after HBO. Additionally, signal intensity was increased in *T*_2_-weighted imaging in frontal white matter and thalamus after NBO as well as in superior sagittal sinus and vitreous body after HBO. FLAIR signal intensities were increased only in the vitreous body after NBO and HBO. In contrast, FLAIR signal of caudate nucleus was decreased after NBO. Statistically significant differences between HBO and NBO were observed in FLAIR signal intensities of caudate nucleus, vitreous body, putamen, frontal white matter, hippocampus and thalamus and also in *T*_1_-weighted signal intensity of vitreous body.

**FIGURE 2.**
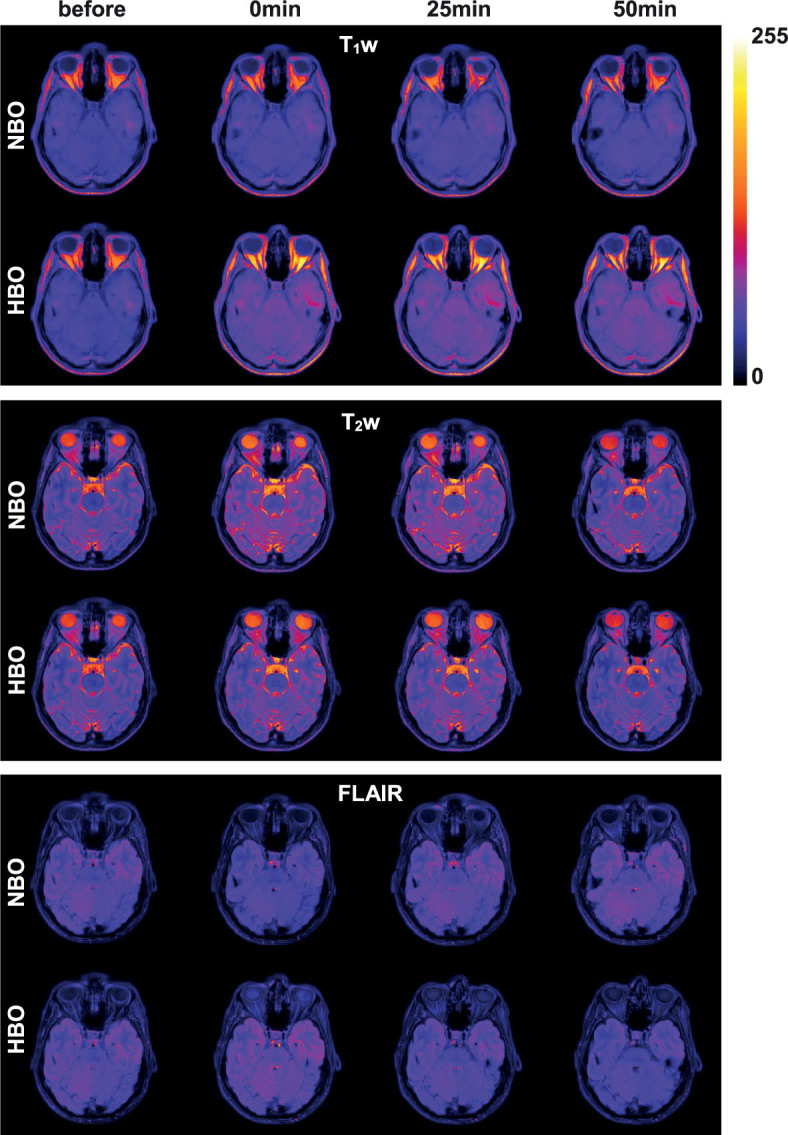
Representative MRI images in healthy subject at baseline, immediately after the end, after 25 min and after 50 min of NBO or HBO.

In our study, *T*_1_-weighted signal intensity of brain structures increased progressively with time after NBO/HBO and was the highest 50 min after the end of both, HBO and NBO. This finding is in agreement with the paramagnetic effect of O_2_. Increased level of dissolved paramagnetic molecular O_2_ shortens *T*_1_*-*relaxation times due to dipoldipol interactions and increases signal intensity on *T*_1_-weighted images.^4,7,8,9,10,11,12,13,14,15,16 23 24^ Relaxation rate (*R*_1_ = 1/*T*_1_) increases proportionally with increasing pO_2_ in inspired gas mixture, the increase being linear or logarithmic when in normobaric or hyperbaric conditions, respectively.^23,24^ The various increase of *T*_1_-weighted signal intensities in the observed tissues might be explained by increased microvascular pO_2_ as well as by differences in tissue oxygenation.^7^ The sustained increase in *T*_1_-weighted signal intensity is further supported by a study of Rockswold *et al*. which showed significantly elevated brain tissue pO_2_ 30 min after the end of HBO and NBO.^25^ In contrast to Rockswold *et al*., we failed to observe a peak in *T*_1_-weighted signal intensity immediately after the end of oxygen therapy. A possible explanation is that the time delay between HBO/NBO and MRI was too long to detect the peak.

We observed progressive increase in *T*_1_-weighted signal intensities after both NBO and HBO along with MRI imaging time, with the highest signal increase at the end of imaging protocol. This phenomenon could not be attributed solely to changes in pO_2_, but also to the effect of ROS on *T*_1_ and *T*_2_-weighted images. Since ROS such as hydroxyl and superoxide radicals contain unpaired electrons, they also exhibit strong paramagnetic effect (a strong *T*_1_ relaxation times shortening) and only a small, statistically insignificant reduction of *T*_2_ relaxation times.^5,6^ Additional point to consider is that distinct neurons respond to oxidative stress differently^26,27^, which leads us to presumption that the effect of HBO-induced oxidative stress would lead to different levels of ROS and thus different effect on *T*_1_ and *T*_2_ weighted signal intensities in various brain regions.

The increase of *T*_1_-weighted signal intensities was more pronounced in frontal white matter and thalamus after HBO compared to NBO. This could be explained by altered O_2_ diffusion after HBO. We observed increased signal intensity in superior sagittal sinus and CSF only after NBO, but not after HBO. Longer time delay between HBO and MRI most likely lowered pO_2_ in the aforementioned fluids before the beginning of MRI. We showed that *T*_1_-weighted signal intensity of vitreous body was significantly increased immediately after the end of HBO exposure and then decreased in subsequent imaging blocks. This is in accordance with expected pO_2_ dynamics in vitreous body, described by Shui *et al*.^28^ Surprisingly, after the exposure to NBO, no increase in vitreous *T*_1_-weighted signal intensity was observed. A possible explanation is that lower vitreous pO_2_ (as achieved during NBO compared to HBO) dropped to baseline level before the beginning of the MRI.

In our study, there were statistical differences in *T*_2_-weighted signal intensities between baseline and after NBO in frontal white matter and thalamus. This is in accordance with Wu *et al*. who observed significant differences in *T*_1_ and *T*_2_ between grey and white matter after inhalation of NBO.^12^ According to Wu *et al.*, *T*_2_ relaxation time increases in rat brain with hyperoxia. In contrast, Tadamura *et al.* did not observe this effect in human “non-brain” tissues (myocardium, spleen, liver, subcutaneous fat, skeletal muscle and bone marrow).^7^ Therefore, it is possible that the effect of hyperoxia on *T*_2_-weighted signal intensities appears to vary in different tissues. In the present study, a significant increase in *T*_2_-weighted signal intensities was also observed after HBO in superior sagittal sinus and vitreous body. Oxygen affects spin-spin relaxation time (*T*_2_) by two competing mechanisms, i.e. *T*_2_ shortening analogous to effect on *T*_1_ (although the effect on *T*_2_ is much smaller) and *T*_2_ lengthening due to diffusion of water protons through field inhomogeneities induced by deoxyhemoglobin generated field gradients (blood-oxygen-level-dependent (BOLD) effect).^13,17^ An increased *T*_2_-weighted signal intensity after NBO and HBO in our study suggests that in human brain structures and vitreous body the paramagnetic effect of oxygen on *T*_2_ relaxation times shortening prevails over BOLD effect.

Our results show statistically significant differences between HBO and NBO were observed in FLAIR signal intensities in different brain structures particularly those that are close to CSF spaces. These results are in accordance with previous studies which showed that in patients receiving 100% NBO elevated pO_2_ leads to incomplete signal suppression of CSF in FLAIR imaging.^29,30^ The elevated pO_2_ most likely favors O_2_ entry into the CSF not through the choroid plexus but directly through the walls of arteries and arterioles on the brain surface.^30^ Since in HBO there is up to 2.5 times higher pO_2_, this effect in FLAIR imaging is more pronounced. We observed increase in FLAIR signal of vitreous body immediately after HBO/NBO exposure and then a subsequent decrease in time – again, this is in accordance with expected pO_2_ dynamics in vitreous body, as described by Shui *et al*.^28^

The results of the present study could have also some clinical implications. Namely, the prolonged intubation induces changes of signal intensities in *T*_1_-weighted and FLAIR images of brain MRI^31^ similar as those observed in our study after NBO. Knowing that prolonged oxygenation induces paramagnetic effects in brain tissues as observed in our study, it is important to take this into account when interpreting brain MRI in intubated patients or in patients after HBO therapy.

The present study has several limitations. First, we failed to show significant differences in MRI signal intensities in brain structures after HBO compared to NBO. It would be expected that brain tissue pO_2_ is significantly higher after HBO compared to NBO^21^ due to higher concentration of dissolved O_2_ during HBO.^19^ The only exception was *T*_1_-weighted signal intensity of vitreous body immediately and 25 min after HBO compared to NBO. One possible explanation is that MRI was performed with time delay of 15 minutes after the end of HBO due to logistics. Perhaps with shorter time delay the peak in *T*_1_-weighted signal intensities could be observed similarly as in the study of Rockswold *et al*.^25^ Additionally, the present study was semiquantitative using clinical head MRI protocol and the next step would be more quantitative approach using *T*_1_ mapping and *T*_2_ mapping. Furthermore, we did not measure brain tissue pO_2_ nor levels of ROS, which would help to explain the observed changes in signal intensities in *T*_1_-weighted and *T*_2_–weighted images. Since our study was performed *in vivo* in a group of volunteers measuring of brain tissue pO_2_ seems rather controversial. We could only measure pO_2_ in arterial blood, however these results do not reflect brain tissue pO_2_ directly. However, according to the reference, at 3 ATA pO_2_ in arterial blood increases to nearly 270 kPa and in tissue to above 53 kPa.^32^ In contrast, in NBO conditions, partial pressure of pO_2_ in the brain is expected to be only between 4 – 6.4 kPa according to study of Meixensberger *et al*.^33^ These values are much lower than during HBO. Therefore, we expected similar tissue pO_2_ differences between HBO and NBO in the present study protocol.

In conclusion, the increased *T*_1_-weighted signal intensities were observed in white and grey brain tissues, brain fluids and vitreous body after NBO as well as HBO, without significant differences between both protocols. In addition, the structure limited and diverse signal intensity increase was observed in *T*_2_-weighted imaging and FLAIR after NBO and HBO. However, the prospective quantitative studies are needed to further clarify the effects of NBO and HBO breathing on MRI in human brain.
